# Supplementary material for: Prevalence and intensity of soil-transmitted helminth infections and associated risk factors among household heads living in the peri-urban areas of Jimma town, Oromia, Ethiopia: A community-based cross-sectional study
Source: PLoS One. 2022 Sep 15;17(9):e0274702. doi: 10.1371/journal.pone.0274702 (PMC9477373; doi:10.1371/journal.pone.0274702)
Supplement: S1 Table — (DOCX) [file pone.0274702.s001.docx]

**S1 Table. Socio-demographic characteristics, sanitation and hygiene practice of the household heads in peri-urban Kebeles in Jimma town, Oromia, Ethiopia, May to July 2021 (n = 376)**

| **Variables** | **Categories** | **Frequency** | **Percent (%)** |
| --- | --- | --- | --- |
| Kebeles | Bore | 108 | 28.7 |
|  | Kofe | 50 | 13.3 |
|  | Hora gibe | 33 | 8.8 |
|  | Jiren | 104 | 27.6 |
|  | Ifabula | 81 | 21.5 |
| Marital status | Married | 321 | 85.3 |
|  | Divorced | 24 | 6.3 |
|  | Widowed/widower | 27 | 7.2 |
|  | Separated | 4 | 1.0 |
| Type of toilet | Flush latrine | 7 | 2.0 |
|  | Traditional pit latrines with slab | 196 | 26.0 |
|  | open pit without slab (no roof) | 139 | 39.7 |
|  | VIP (ventilated improved pit latrines | 8 | 2.3 |
| Drinking water | Tap water | 126 | 35.0 |
|  | Tube hole/borehole | 65 | 17.3 |
|  | Protected well/spring | 174 | 46.3 |
|  | Unprotected hole/spring | 11 | 2.9 |
| Water for domestic use | Tap water | 73 | 19.4 |
|  | Tube hole/borehole | 66 | 17.6 |
|  | Protected well/spring | 194 | 51.6 |
|  | Unprotected hole/spring | 35 | 9.3 |
|  | Surface water | 8 | 2.1 |
| Solid wastes disposal | Open disposal | 254 | 67.6 |
|  | Solid waste pit | 42 | 11.2 |
|  | Open burn | 80 | 21.3 |
| Liquid wastes disposal | Drain directly to the garden | 356 | 94.7 |
|  | Discharge in to street surface | 13 | 3.5 |
|  | Liquid waste pit | 7 | 1.9 |

* small administrative unit in Ethiopia with about 5000 household population
