# Supplementary material for: Prevalence and intensity of soil-transmitted helminth infections and associated risk factors among household heads living in the peri-urban areas of Jimma town, Oromia, Ethiopia: A community-based cross-sectional study
Source: PLoS One. 2022 Sep 15;17(9):e0274702. doi: 10.1371/journal.pone.0274702 (PMC9477373; doi:10.1371/journal.pone.0274702)
Supplement: S2 Table — (DOCX) [file pone.0274702.s002.docx]

**S2 Table. Prevalence of STH and its distribution by socio-demographic characteristics, sanitation and hygiene practice of the household heads in peri-urban Kebeles in Jimma town, Oromia, Ethiopia, May to July 2021 (n = 376)**

| **Variables** | **Categories** | **STH prevalence** | |
| --- | --- | --- | --- |
|  |  | **Positive n (%)** | **Negative n (%)** |
| Kebeles^*^ | Bore | 30(27.8) | 78(72.2) |
|  | Kofe | 9(18) | 41(82) |
|  | Hora gibe | 4(12.1) | 29(87.9) |
|  | Jiren | 11(10.6) | 93(89.4) |
|  | Ifabula | 14(17.3) | 67(82.7) |
| Marital status | Married | 63(19.6) | 258(80.4) |
|  | Divorced | 2(8.3) | 22(91.7) |
|  | Widowed/widower | 3(11.1) | 24(88.9) |
|  | Separated | 0(0.0) | 4(100) |
| Type of toilet | Flush latrine | 0(0.0) | 7(100) |
|  | Traditional pit latrines with slab | 22(11.2) | 174(88.8) |
|  | open pit without slab (no roof) | 37(26.6) | 102(73.4) |
|  | VIP (ventilated improved pit latrines | 1(12.5) | 7(87.5) |
| Drinking water | Tap water | 24(19.0) | 102(81.0) |
|  | Tube hole/borehole | 17(26.2) | 18(73.8) |
|  | Protected well/spring | 26(14.9) | 148(86.1) |
|  | Unprotected hole/spring | 1(9.1) | 10(90.9) |
| Water for domestic use | Tap water | 15(20.5) | 58(79.5) |
|  | Tube hole/borehole | 17(25.8) | 49(74.2) |
|  | Protected well/spring | 31(16.0) | 163(84.0) |
|  | Unprotected hole/spring | 1(2.9) | 34(97.1) |
|  | Surface water | 4(50.0) | 4(50.0) |
| Solid wastes disposal | Open disposal | 41(16.2) | 213(83.8) |
|  | Solid waste pit | 8(19.0) | 34(81.0) |
|  | Open burn | 19(23.8) | 61(76.2) |
| Liquid wastes disposal | Drain directly to the garden | 63(17.7) | 293(82.3) |
|  | Discharge in to street surface | 2(15.4) | 11(84.6) |
|  | Liquid waste pit | 3(42.9) | 4(57.1) |

* small administrative unit in Ethiopia with about 5000 household population
